# Tuning the Loading and Release Properties of MicroRNA-Silencing Porous Silicon Nanoparticles by Using Chemically Diverse Peptide Nucleic Acid Payloads

**DOI:** 10.1021/acsbiomaterials.1c00431

**Published:** 2021-09-01

**Authors:** Martina Neri, Jinyoung Kang, Jonathan M. Zuidema, Jessica Gasparello, Alessia Finotti, Roberto Gambari, Michael J. Sailor, Alessandro Bertucci, Roberto Corradini

**Affiliations:** † Department of Chemistry, Life Sciences and Environmental Sustainability, University of Parma, Parco Area delle Scienze 17/A, 43124 Parma, Italy; ‡ Department of Nanoengineering, University of California San Diego, 9500 Gilman Drive, La Jolla, California 92093, United States; § Department of Chemistry and Biochemistry and Department of Neurosciences, University of California San Diego, 9500 Gilman Drive, La Jolla, California 92093, United States; ∥ Department of Life Sciences and Biotechnology, University of Ferrara, Via Fossato di Mortara 74, 44121 Ferrara, Italy; ⊥ Department of Chemistry and Biochemistry, University of California San Diego, 9500 Gilman Drive, La Jolla, California 92093, United States

**Keywords:** oligonucleotide mimics, drug delivery, anti-microRNA therapeutics, nanomaterials, release kinetics

## Abstract

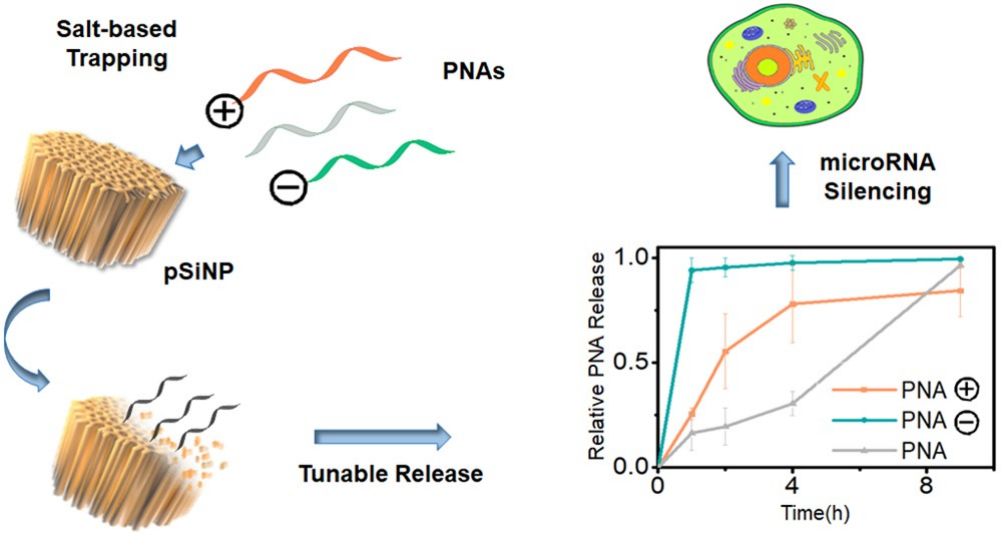

Peptide nucleic acids (PNAs) are a class of artificial oligonucleotide mimics that have garnered much attention as precision biotherapeutics for their efficient hybridization properties and their exceptional biological and chemical stability. However, the poor cellular uptake of PNA is a limiting factor to its more extensive use in biomedicine; encapsulation in nanoparticle carriers has therefore emerged as a strategy for internalization and delivery of PNA in cells. In this study, we demonstrate that PNA can be readily loaded into porous silicon nanoparticles (pSiNPs) following a simple salt-based trapping procedure thus far employed only for negatively charged synthetic oligonucleotides. We show that the ease and versatility of PNA chemistry also allows for producing PNAs with different net charge, from positive to negative, and that the use of differently charged PNAs enables optimization of loading into pSiNPs. Differently charged PNA payloads determine different release kinetics and allow modulation of the temporal profile of the delivery process. *In vitro* silencing of a set of specific microRNAs using a pSiNP-PNA delivery platform demonstrates the potential for biomedical applications.

Peptide nucleic acids (PNAs) are artificial oligonucleotide mimics that have gathered much attention over the last decades for their potential as therapeutic agents and as tools for molecular diagnostics.^1,2^ The absence of electrostatic charge due to a N-(2-aminoethyl)glycine backbone makes the hybridization of PNAs with complementary DNA or RNA strands more favorable than that between natural oligonucleotides.^3^ The presence of this unnatural backbone is also why PNAs have exceptional biological stability: in contrast to natural nucleic acids and peptides, they are not recognized and degraded by nucleases and proteases. Another crucial feature of PNAs is that by using standard solid-phase manual or automated synthesis^4^ typical of peptide synthesis, PNA monomers can be easily assembled and conjugated with a range of diverse functional groups, such as fluorophores, amino acids, or small molecules. These properties have all been harnessed to exploit the use of PNAs in a wide range of biomedical applications, from precision medicine therapeutics to chemical biology to molecular diagnostics.^5,6^ However, the uncharged nature of the PNA backbone also generates limitations—it can result in low water solubility compared to DNA and RNA and low cellular internalization in drug delivery applications. Several strategies to overcome these issues have been pursued.^7^ Some of the most promising approaches involve conjugation with cell-penetrating peptides,^8−12^ or formulation with liposomes,^13−15^ polymer nanoparticles,^16−21^ and carbon-based nanocarriers.^22−25^ Porous inorganic nanocarriers have also been demonstrated to be particularly suited for the loading and the delivery of PNA.^26−28^ Among these, porous silicon nanoparticles (pSiNPs) have emerged as a class of nanomaterials with a suite of properties that makes them especially amenable to accommodating and releasing biomolecular payloads. The intrinsic photoluminescence, the biocompatibility, and the biodegradability of porous silicon structures enable their use as drug delivery platforms for *in vivo* applications.^29−31^ Porous silicon nanomaterials are fabricated via electrochemical anodization of single crystalline silicon wafers in hydrofluoric acid solution. The advantage of this electrochemical synthesis procedure lies in the high degree of control it exerts over the morphological properties of the pSi material, such as pore size, thickness, and porosity, simply by varying the experimental and instrumental parameters.^32^ In addition, the surface of porous silicon materials, which naturally tends to present a thin layer of silicon oxide if left untreated after synthesis, can be conveniently modified with different groups and molecules by exploiting currently available strategies based on silane chemistry. This also plays a key role in regulating the dissolution rate of pSiNPs in aqueous media and therefore in the release of payloads in biological settings. All these features make pSiNPs attractive delivery vehicles for PNA. However, interfacing PNA with pSiNPs is not a trivial task, because the uncharged nature of PNA prevents the use of conventional loading strategies based on electrostatic adsorption of anionic oligonucleotides. In 2014, a first work explored *in situ* synthesis of PNA on pSi supports and showed that this strategy could afford a loading value of 8.6 × 10^–4^ mol PNA/g pSi and a sustained release in aqueous buffer.^33^ These pSiNP-PNA complexes enabled the delivery of specific anti-microRNA (anti-miR) PNA sequences in human hepatic carcinoma cells. Beavers et al. deployed an endosomolytic two-block polymer as a coadjuvant for the encapsulation of PNA in pSiNPs. This approach allowed highly efficient loading of PNA, and polymer-pSiNP composites were used to deliver anti-microRNA PNA sequences *in vivo*.^34^ However, using a polymer-based formulation in tandem with pSiNPs requires additional synthetic effort and an increase in the number of components in the final formulation, which may hinder clinical translation. In this study, we establish a simpler and more expedited means to encapsulate PNA in pSiNPs that achieves highly efficient loading and enables fine-tuning of the temporal properties of payload release. Our approach leverages the chemical versatility of PNA, which, different from other oligonucleotide analogues, can be synthesized with a user-defined net charge, and a single-step salt-based loading technique that requires neither adjuvants nor derivatization reactions at the nanoparticle surface. Here, we encapsulated a small library of differently charged PNAs into pSiNps and demonstrated that the chemical–physical nature of the PNA payload determines its release kinetics in aqueous buffer. Importantly, we show that pSiNPs-PNA complexes fabricated in this way hold promise as a nanomedicine platform, as we demonstrate their use *in vitro* for the delivery of anti-miR PNAs in human bronchial epithelial cells.

Recently, a new procedure for loading of small interfering RNA (siRNA) into pSiNPs was developed in the Sailor group. This is based on physical trapping of the synthetic oligonucleotides in the pores of the particles caused by the simultaneous precipitation of an insoluble shell of calcium silicate Ca_2_SiO_4_ at the particle surface.^35^ This method enabled loading of a considerable amount of siRNA in the particles in the range 20–25% by mass and allowed efficient payload release *in vivo*. The presence of the calcium silicate coating causes a slower release of the siRNA payload compared to formulations based on electrostatic interactions, because the pSi skeleton is prevented from an early dissolution in aqueous media.^35^ The same method was then employed for the loading and delivery of synthetic LNA,^36^ DNA,^37^ and RNA payloads,^38−40^ which all capitalize on the negatively charged phosphate-based backbone of the synthetic oligonucleotides. Motivated by the simplicity and the efficacy of this approach, we set out to explore its extension to PNA payloads. For this, we have engineered a small library of PNAs with different net charge, which would allow us to systematically study how varying this property could modulate the efficiency of the above trapping process. For this study, we have selected three different PNA sequences: PNA1: H-TTTCGTTATTGCTCTTGA-Gly-NH_2_, PNA2: H-AGTTATCACAGTACTGTA-Gly-NH_2_, PNA3: H-AGGGATTCCTGGGAAAAC-Gly-NH_2_. These sequences are complementary to three specific microRNAs, i.e., miR-335, miR-101, and miR-145, respectively, and may be potential candidates in microRNA-silencing therapeutic strategies for cystic fibrosis.^41−43^ PNAs can be easily modified and diversified by introducing chemical variations at both the backbone and the nucleobase level or through the conjugation with amino acids along the strand. To obtain a set of differently charged PNAs, we conjugated each sequence at the N-terminus to either eight arginine (PNA-R8) or glutamic acid residues (PNA-E8) (Figure 1A). The synthesis was carried out manually following a standard Fmoc-based solid-phase synthesis (SPS) protocol, and the products were all purified by RP-HPLC and characterized by means of ESI-MS (Figure S1–S9, Supporting Information, SI). PSiNPs were produced by electrochemical etching of highly boron-doped p^+2^ single-crystal silicon wafers in a hydrofluoric acid electrolyte following a well-established “perforation” procedure.^32^ The as-etched nanoparticles were characterized by transmission electron microscopy (TEM) (Figure 1D), and their hydrodynamic radius was found to be 215 ± 8 nm by dynamic light scattering (DLS) (Figure S10A, SI). The porous layer porosity was measured to be 58% ± 4% by a nondestructive spectroscopic liquid infiltration method (SLIM).^44^ Cryogenic nitrogen adsorption isotherm using the Brunauer–Emmett–Teller (BET) method allowed determination of a total pore volume of 1.33 cm^3^ g^–1^, a surface area of 370.5 m^2^ g^–1^, and an average pore size of 14.5 nm (Figure S12). Such pore size, together with the above particle size, is well-fit for accommodating a macromolecule such as PNA. Oxidized pSiNPs were then loaded with the differently charged PNAs following a calcium silicate trapping procedure in the presence of a high concentration (4 M) of calcium chloride (CaCl_2_) (Figure 1B). This procedure allows mechanical entrapment of the oligonucleotides within the pores of the particles due to the precipitation of calcium silicate Ca_2_SiO_4_ at the nanoparticle surface (Figures 1E and S11, SI). The three formats of neutral, negative, and positive PNA were, respectively, added (final concentration 15 μM) to a dispersion of pSiNPs in ethanol (1 mg/mL) in the presence of CaCl_2_ and incubated for 45 min. Calcium silicate-based trapping of PNA led to a slight increase in the average hydrodynamic diameter of the nanoparticles (up to ∼270 nm) and a shift in the ζ-potential to less negative values (Figure 1C). Interestingly, we registered negative values of ζ-potential for all three classes of PNA. This suggests that the trapping procedure results in an efficient encapsulation of the different PNA payloads in the nanoparticle pores, and the final surface charge of the loaded nanoparticles is only determined by the presence of the calcium silicate coating formed. This is particularly evident in the case of positively charged PNA (Figure 1C). PNA loading (defined as the mass of the oligonucleotide divided by the total mass of the oligonucleotide + Si nanoparticles) was quantified by UV–vis spectroscopy measurements of the supernatants at λ = 260 nm.

**Figure 1:**
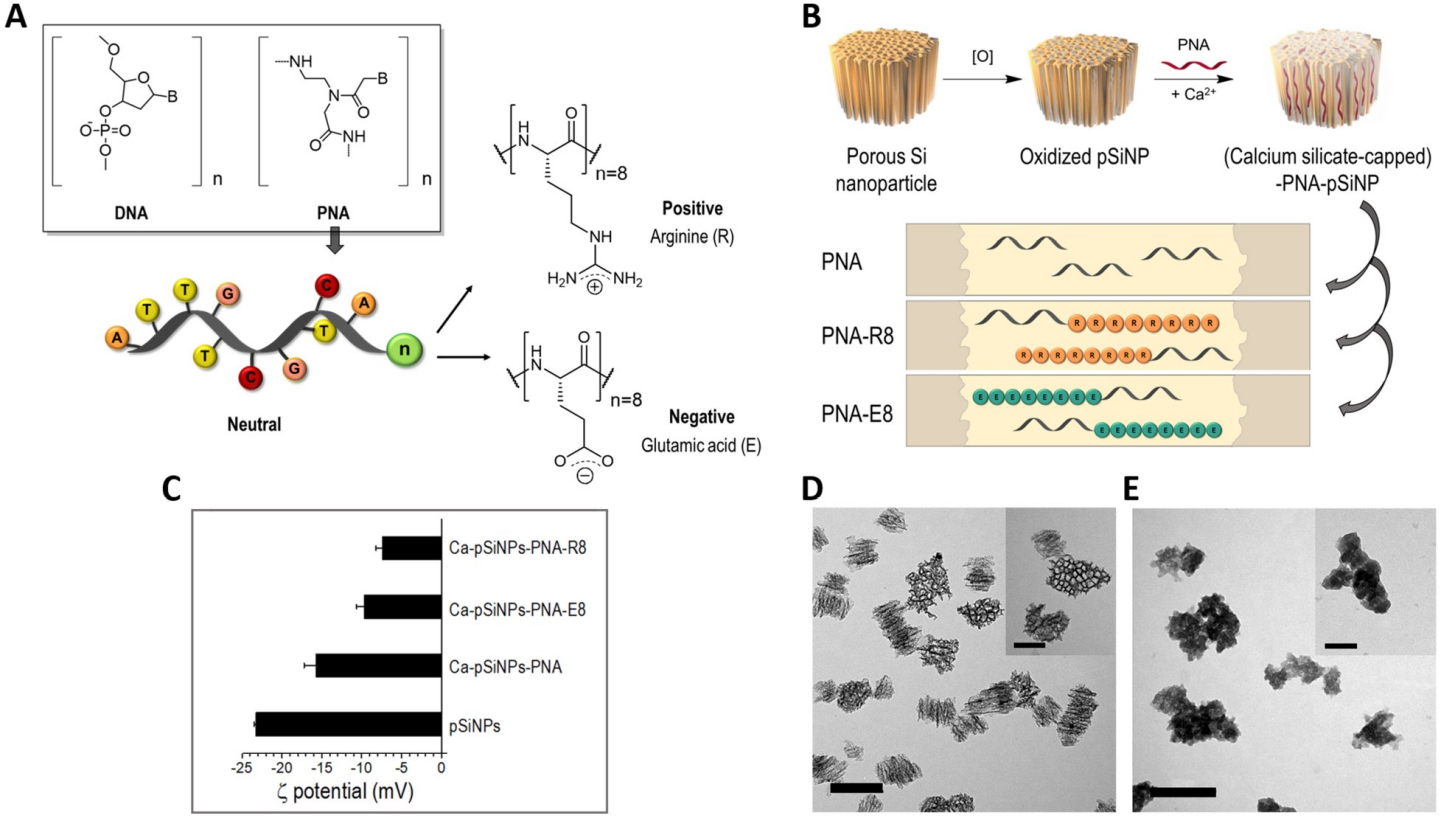
Preparation and characterization of Ca-pSiNPs-PNA. (A) Pictorial representation of a neutral PNA oligomer that can be conjugated to differently charged amino acid residues. (B) Schematic representation of the calcium silicate trapping protocol used to produce PNA-loaded pSiNPs. (C) ζ-Potential measurements of oxidized pSiNPs and pSiNPs loaded with PNA, PNA-E8, and PNA-R8, respectively, following salt-based trapping using calcium silicate (mean ± SD, *n* = 3). (D) Transmission electron microscope (TEM) image of freshly etched pSiNPs (scale bar = 200 nm); the inset shows a close-up of a single particle (scale bar = 100 nm). (E) TEM image of calcium silicate-capped pSiNP-PNA complexes (scale bar = 200 nm); the inset shows a close-up of a single particle (scale bar = 100 nm).

The loading values were calculated to be ∼17% w/w for all the positively charged (PNA_*X*_-R8) and negatively charged (PNA_*X*_-E8) PNAs tested, irrespective of their sequence composition. The use of neutral PNAs led to a lower loading of ∼8% w/w. This result could be ascribed to the complete lack of charge in the neutral PNA, which may determine a lower solubility in the working solution and/or may not provide any significant driving force to the encapsulation process, as no electrostatic interactions occur between the oligonucleotide, the nanoparticles, and the calcium-pSi layer. The loading values calculated for the entire set of PNA payloads tested are reported in Table 1, where loading is also expressed in terms of nanomoles of PNA divided by milligrams of pSiNPs. Beavers et al. reported loading values of 20 nmol/mg for neutral PNA in pSiNP–polymer nanocomposite.^34^ In this present case, PNA loading of ∼30 nmol/mg and ∼29 nmol/mg was achieved with negatively charged and positively charged PNAs, respectively, without the use of a coadjuvant polymer. We also note that our method enables loading of neutral, unmodified PNA in pSiNPs in a very simple way, although with a lower efficiency when compared to charged PNAs. The above loading values correspond to loading efficiencies (PNA nmol encapsulated/PNA nmol incubated) of 98.7% ± 0.4%, 96% ± 3%, and 64% ± 2% for PNA-E8, PNA-R8, and PNA, respectively (Figure S13, SI). Next, we systematically studied the release kinetics of pSiNP systems loaded with differently charged PNAs. Release of PNA from the calcium silicate-sealed nanoparticles was achieved by incubation in aqueous phosphate buffered saline (PBS), pH 7.4 at 37°, which simulates physiological conditions. The concentration of PNA released into the solution at specific time points was determined by measuring its UV–vis absorbance intensity (λ = 260 nm). Three distinct release profiles were obtained based on the net charge of the PNA payload. The release profile of the anionic PNA-E8 (Figure 2A) is characterized by a rapid kinetics, leading to a burst release of ∼95% of the total PNA-E8 loaded within the nanoparticles in the first 2 h. This is comparable to the degradation profile reported for Ca-pSiNPs loaded with locked nucleic acid (LNA) and siRNA.^35,36^ Nearly 100% efficiency release (defined as mass of released PNA divided by mass of loaded PNA) was observed in this case (Figure S13, SI). PSiNPs loaded with cationic PNA-R8 showed a slower release kinetics compared the previous pSiNP-PNA-E8-complexes (Figure 2B,D,E). In this case, nearly 80% of the loaded PNA-R8 was released within the first 8 h and 95% over 24 h (Figure 2B). Nearly quantitative release of the PNA-R8 payload was observed within 24 h (Figure S13, SI). The release kinetics of pSiNPs loaded with neutral PNA showed another different, slower temporal profile compared to PNA-R8 and PNA-E8. No major burst release was observed in this case, with an average of 55% of the total PNA loaded from Si nanoparticles released in the first 8 h. We assume that the absence of electrostatic interactions between the nanoparticles and the oligonucleotide cargo may play a crucial role in its release kinetics. Contrary to the first two cases, the neutral PNA payload was released with an efficiency of ∼64% (Figure S13, SI). It is possible that the absence of electrostatic interactions due to the neutral nature of the PNA payload may cause a slower release kinetics and a more difficult degradation process when compared to the use of negatively or positively charged PNAs. Ion exchange processes normally contribute to the release of charged payloads from nanoparticle carriers.^26,45^ In addition, all the final Ca-pSiNPs-PNA complexes displayed a net negative charge on their surface as demonstrated by the ζ-potential measurements, and this may accelerate the release kinetics of negatively charged PNA because of electrostatic repulsion at the interface once the degradation of the particles has started. Electrostatic interactions and ion exchange mechanisms are missing when using a neutral PNA, which may cause the release kinetics to be exclusively associated with the degradation of the nanoparticles. The distinct release rate characterizing the three different formulations is demonstrated by the release efficiency (PNA released/PNA loaded) observed after 2 h in each specific case, which shows that pSiNPs release the anionic payload PNA-E8 2-fold and ∼7-fold faster than cationic PNA-R8 and neutral PNA, respectively (Figure 2E). The difference in the release rate of the three formulations suggests that PNA delivery tuned in time may be achieved by playing on the net charge of the specific PNA payload used. To expand the application window of our method, we also demonstrated that combined loading and release of two PNAs with different sequences is readily obtainable. As a proof-of-principle demonstration, we set out to coload and release two PNAs bearing different sequences. A preliminary experiment was performed using the two negative PNAs PNA1-E8 and PNA2-E8, which were simultaneously coloaded in the particles following the same trapping protocol.

**Table 1:** Loading of Different PNA Payloads

PNA payload	loading (nmol/mg)	loading value (%)
PNA 1- R8	29 ± 3	17.6
PNA 1- E8	30 ± 1	17.7
PNA 1	16.4 ± 0.8	8.7
PNA 2- R8	27 ± 2	16.7
PNA 2- E8	29.7 ± 0.3	17.7
PNA 2	16 ± 4	8.7
PNA 3- R8	27 ± 2	16.7
PNA 3- E8	29.2 ± 0.7	17.7
PNA 3	16 ± 4	8.8

**Figure 2:**
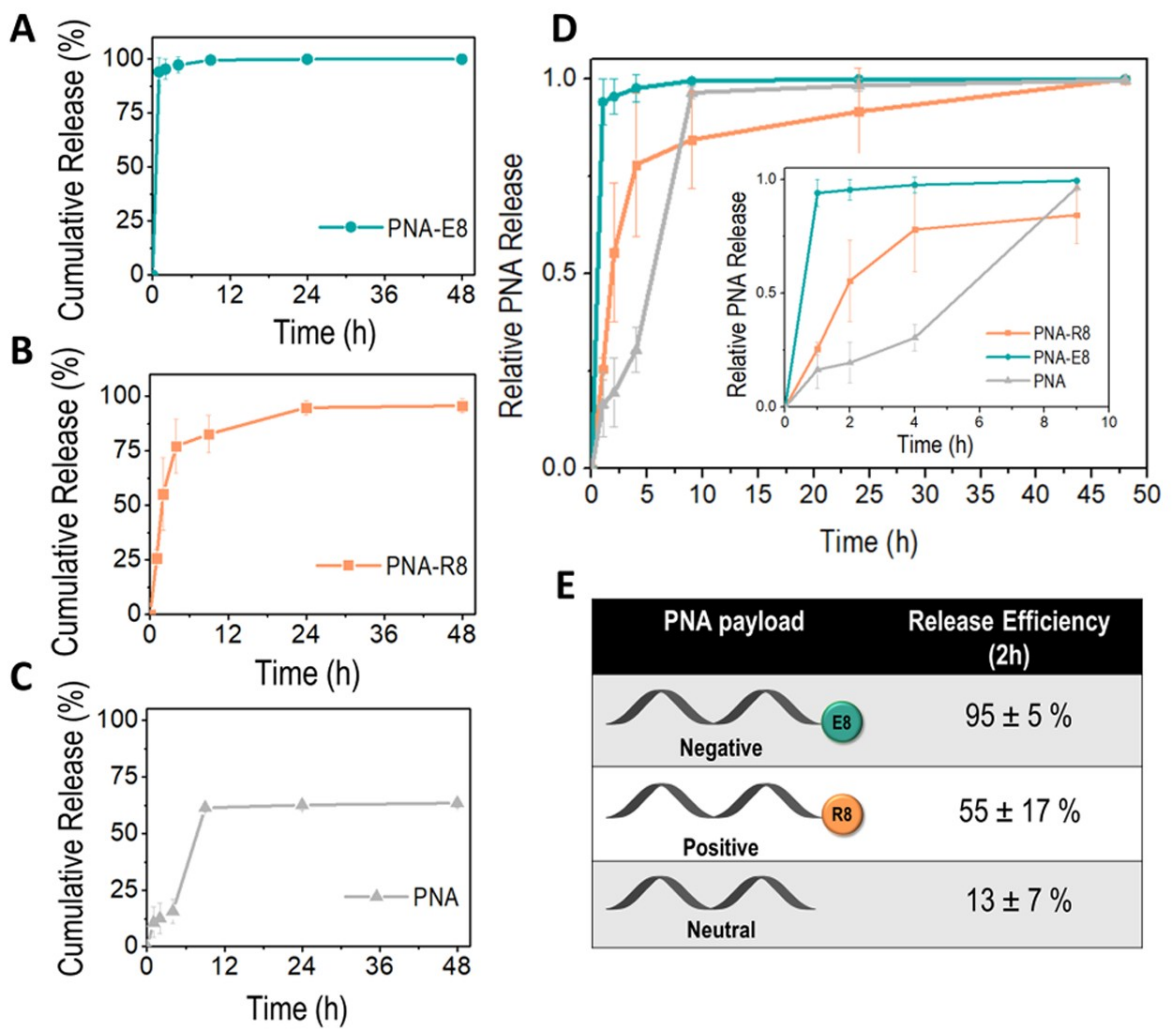
Release kinetics of pSiNPs loaded with differently charged PNA payloads. Cumulative release % of PNA-E8 (A), PNA-R8 (B), and PNA (C) over 48 h (mean ± SD, *n* = 3). The fractional quantity of PNA released from pSiNPs shows a faster release kinetics for anionic PNA-E8 followed by cationic PNA-R8 and neutral PNA. Relative PNA release was normalized to the same scale, where 1.0 equals to the total amount of PNA released in 48 h (D). Three distinct release rates are expressed as release efficiency (defined as mass of released PNA divided by mass of loaded PNA) at the 2 h time point (E).

The loading value (calculated as the mass of the oligonucleotides PNA1-E8 + PNA2-E8 divided by the total mass of the PNAs + Si nanoparticles) was comparable to that found for a single-PNA loading (∼17%). Release of PNA payloads was carried out in PBS, pH 7.4 at 37° over 24 h. The presence of both species in the solution was confirmed by UPLC-ESI-MS analysis of the supernatant, which demonstrated that after the first 2 h both PNAs had already started to be released (Figure S14, SI). This is important because porous silicon nanoparticles loaded with a combination of PNA-based therapeutics may enable synergistic strategies targeted to multiple biological pathways at once. Next, to demonstrate the potential of our formulation in drug delivery applications, we decided to investigate cell internalization of calcium silicate-capped pSiNP-PNA complexes and their ability to recognize and silence cognate microRNA targets. In this study, the PNA sequences PNA1, PNA2, and PNA3 were selected for their potential as anti-miR therapeutics in the context of cystic fibrosis, an autosomal recessive genetic disease deriving from misfunctioning of cystic fibrosis transmembrane conductance regulator (CFTR) protein. Inhibition of specific microRNAs involved in the regulation of the CFTR gene has been proposed as a potential therapeutic approach.^41−43,46,47^ For this application, we set out to test a neutral PNA payload because that represents the most challenging one to internalize in cells. As mentioned earlier in the text, the lack of charge in the canonical PNA backbone results in very poor cellular internalization properties. Conversely, positively charged PNAs have been shown to display quite effective cell internalization due to interaction with the negatively charged cell membrane. Neutral PNAs are also the most difficult to noncovalently load into inorganic nanoparticles, such as pSiNPs, because any method based on the use of electrostatic interactions is not feasible. In contrast, negatively charged PNAs can be integrated in nanoparticle systems relying on the variety of methods available for DNA or RNA oligonucleotides. Motivated by these arguments, we prepared a set of pSiNPs loaded with PNA1, PNA2, and PNA3, respectively, following the salt-based trapping method established above (Figure 3A). In this case, PNA strands were synthesized with rhodamine B (Rho) conjugated at one terminus, which allowed for obtaining fluorescent PNAs useful for fluorescence-based cell internalization studies. Loading into pSiNPs was performed according to the aforementioned protocol and gave analogous loading values. Cellular uptake of the three different pSiNP-PNA complexes was studied *in vitro* using a human bronchial epithelial IB3–1 cell line as a cellular model. IB3–1 cells were incubated for 24 h with a concentration of nanoparticles that, based on the calculated loading value, translated in either a 2 × 10^–6^ M or a 4 × 10^–6^ M dose of PNA (PNA1, PNA2, PNA3, respectively). Following treatment, cellular internalization was evaluated by means of fluorescence-activated cell sorting (FACS) analysis, which showed effective internalization of the pSiNP-PNA complexes in all three cases based on the fluorescence signal due to rhodamine B (Figures 3B and S15 and S16, SI). Internalization of pSiNP-PNA complexes and delivery of PNA were further studied by imaging living cultured cells by means of a BioStation IM (Nikon, Minato, Tokyo, Japan). A representative set of results is shown in Figure 3C. Neither intracellular uptake nor PNA distributed in the cytoplasm could be observed in cells cultured with only PNA1 not incapsulated in pSiNPs (Figure 3C, panel c). On the contrary, almost all the cells cultured in the presence of pSiNP-PNA complexes displayed internalization and distribution of PNA in the cytoplasm (Figure 3C, panels d–g). A complete set of results obtained for the different pSiNP-PNA1, pSiNP-PNA2, and pSiNP-PNA3 complexes is reported in Figure S17 in the SI.

**Figure 3:**
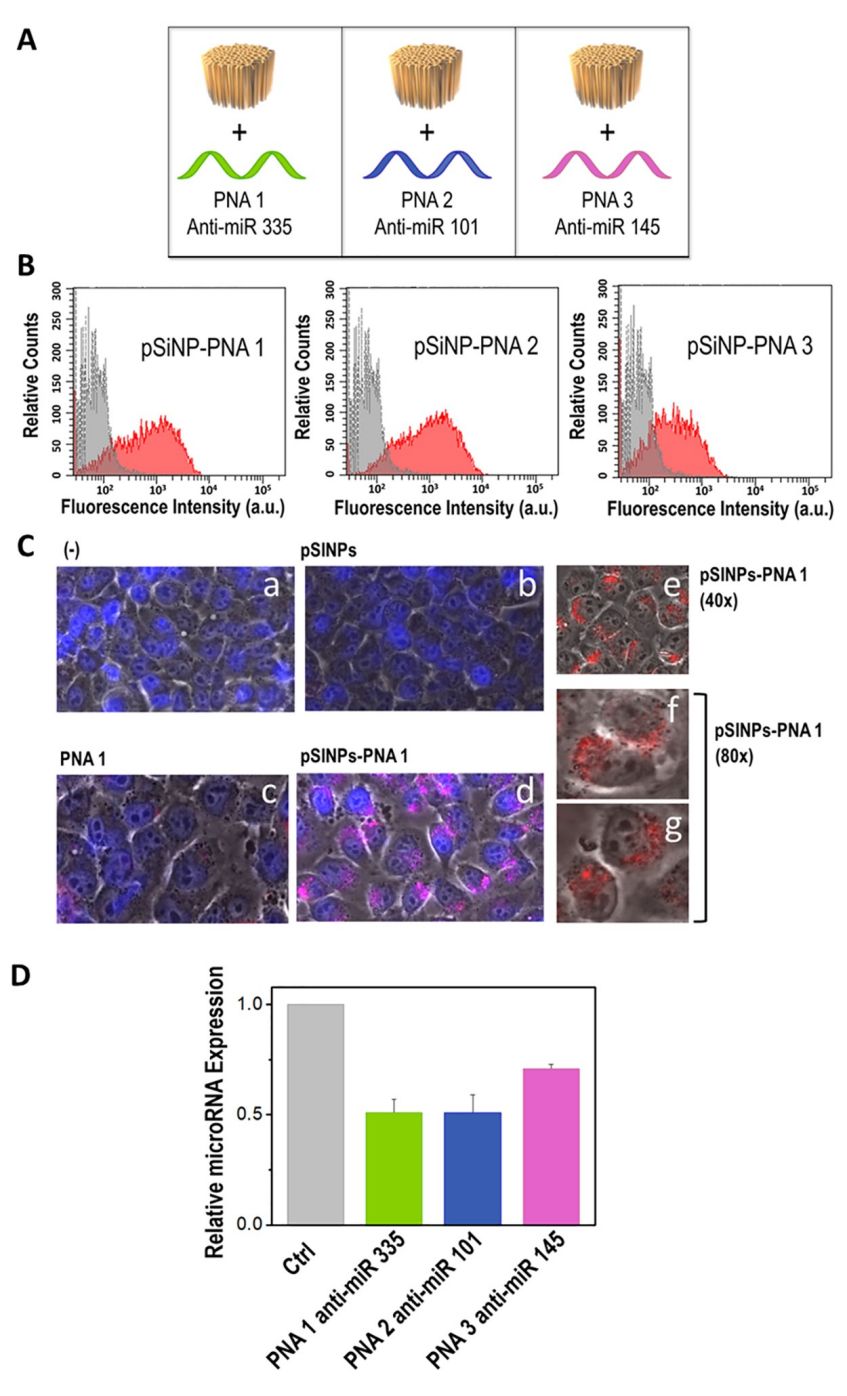
Investigation of cellular uptake and microRNA silencing of PNA 1, PNA 2, and PNA 3 trapped into pSiNPs. (A) Schematic drawing of the three distinct pSiNPs-PNA complexes tested in biological assays. (B) Flow cytometry graphs of untreated IB3–1 cells (gray) and IB3–1 cells treated with doses of pSiNPs corresponding to 4 μM PNA (red). The fluorescent signal derives from the rhodamine B tag of the PNA payloads. (C) Internalization evaluated using BioStation IM (Nikon, Minato, Tokyo, Japan). Cells were either untreated (a) or treated with pSiNPs (b), only PNA1 (c), or pSiNP-PNA1 complexes (d). Pictures are presented as a merger of live, DAPI, and TRITC images. Higher-magnification images showing the cellular distribution of PNA when using pSiNP-PNA1 complexes are obtained as a merger of live and TRITC images (e–g). (D) Relative microRNA expression evaluated by reverse transcriptase quantitative polymerase chain reaction (RT-qPCR) for miR-335, miR-101, and miR-145 in IB3–1 cells treated with pSiNPs carrying PNA 1 anti-miR-335, PNA 2 anti-miR-101, and PNA 3 anti-miR-145, respectively (mean ± SEM, *n* = 4).

The ability of each PNA sequence released in the cytosol to bind and silence its cognate target microRNA was then measured by quantitative reverse transcription polymerase chain reaction (RT-qPCR). Incubation of independent samples of IB3–1 cells with each of the three different pSiNP-PNA complexes, respectively, led to a reduction of the expression (bioavailability) of the cognate microRNA target of ∼50% in the case of miR-335 and miR-101, and of ∼30% in the case of miR-145, relatively to untreated control samples. This demonstrates the high versatility of our approach in terms of inducible biological effect. Different PNA sequences targeting a pool of diverse microRNAs were all efficiently delivered to cultured epithelial cells and provided silencing of the desired, specific microRNA target. These results also suggest that even the simplest formats of calcium silicate-capped pSiNP-PNA complexes, i.e., bearing no surface functionalization with targeting ligands or polymer coatings and encapsulating a neutral PNA, are effectively uptaken by cells *in vitro*, and the released PNA payload in the cytosol can bind to and silence its target complementary RNA sequence.

In conclusion, we have demonstrated that PNA can be efficiently loaded into porous silicon nanoparticles following a simple, rapid approach based on calcium silicate trapping. The versatile chemistry of PNA allows for synthesizing oligonucleotides with custom net charge, and this can be used to optimize the efficiency of the trapping process. We have shown that calcium silicate trapping is compatible with both a negative, positive, and neutral PNA payload, and that charged PNAs can be encapsulated with loading values of ∼30 nmol/mg pSi and with an efficiency of >90%. The charge of the PNA payload then determines the release kinetics of the nanoparticle system, with the release rate increasing from a neutral PNA payload (slowest release) to a negative PNA payload (fastest release).

This hints at the possibility to modulate the temporal delivery window of a specific PNA payload by playing on its chemical nature, which may be useful for both therapeutic and synthetic biology applications. The encapsulation of the eight arginine PNA (PNA-R8) into Ca-pSiNPs suggests that other positively charged payloads can be easily incorporated into pSiNPs, which is ideal for minimizing the typical cytotoxicity associated with cationic drugs.^48−50^ In addition, using a calcium silicate-based approach for encapsulation of negatively charged payloads, such as siRNA or other therapeutic oligonucleotides, allows for bypassing the need for cationic polymers that are often proposed for loading anionic payloads in nanoparticle carriers through electrostatic interaction and that raise concern for their potential cytotoxicity.^35,40,51^ PSiNPs loaded with neutral PNA payloads successfully delivered their cargo in a model cell line relevant to cystic fibrosis and enabled silencing of three specific target microRNAs. Delivery of CFTR-upregulating anti-miRNA PNAs can support new therapeutic strategies for the treatment of cystic fibrosis.^52,53^ These results all demonstrate a strategy for formulating PNA into pSiNPs for therapeutic applications in precision medicine. Further engineering of the nanoparticle carrier, including functionalization with targeting elements, such as peptides, and/or surface PEGylation, and rationalization of the PNA payload, e.g., combining differently charged PNAs in the same nanoparticle to achieve staggered delivery in time, will help expand the applicability window and foster the therapeutic uses of PNA-pSiNP complexes as nanomedicine formulations.

## Supporting Information Available

Description of the materials and methods and results related to PNA characterization, pSiNPs fabrication, release studies, cellular uptake assay, and coloading assay (PDF)
