# Supplementary material for: Tuning the Loading and Release Properties of MicroRNA-Silencing Porous Silicon Nanoparticles by Using Chemically Diverse Peptide Nucleic Acid Payloads
Source: ACS Biomater Sci Eng. 2021 Sep 1;8(10):4123–31. doi: 10.1021/acsbiomaterials.1c00431 (PMC9554869; doi:10.1021/acsbiomaterials.1c00431)
Supplement: Supplementary file 1 — ab1c00431_si_001.pdf [file ab1c00431_si_001.pdf]

Supporting Information

Tuning the loading and release properties of microRNA-silencing porous silicon nanoparticles by using chemically diverse peptide nucleic acid payloads

Martina Neri, Jinyoung Kang, Jonathan M. Zuidema, Jessica Gasparello, Alessia Finotti, Roberto Gambari, Michael J. Sailor, Alessandro Bertucci*, Roberto Corradini.

1. Materials and Methods

2. Supporting Figures

1. Materials and Methods

PNA Synthesis: All the PNAs were synthesized at the 5 μmol scale using standard manual Fmoc-based chemistry with HBTU/DIPEA coupling on a Rink amide resin loaded with Fmoc-Gly-OH as first monomer (0.2 mmol/g). Commercially available Fmoc-PNA(Bhoc)-OH monomers and Rhodamine B were used for the synthesis and conditions adapted form procedures described elsewhere.[1] A Fmoc-based protocol was conducted that involved the following steps: a) deprotection 2x8min ; b) DCM Wash; c) dry DMF Wash; d) Kaiser test (1 min, should be positive); e) coupling 1x30 min (activation 2 min, activation solution: 5 eq of monomer and activator, 10 eq of DIPEA in dry DMF); f) DMF wash; Kaiser test (1min, should be negative); g) capping 2x1min; h) DMF wash; i) DIPEA wash 2x2 min; l) DMF wash; m) DCM wash. The following solutions were used for the solid-phase synthesis of PNA: deprotection: 20% piperidine in DMF; capping: acetic anhydride/DIPEA/dry DMF 5/6/89; DIPEA wash: 5% DIPEA in DMF.
Cleavage of the PNA from the resin was performed by treatment with a TFA/m-cresol (9:1 v:v) solution (2 x 1h); the resin was eliminated by filtration and the filtrate was treated to precipitate PNA by adding at least 10 volumes of diethyl ether. The purity and identity of the PNAs were evaluated by UPLC-ESI/MS, using columns and gradient described elsewhere.[2] The PNAs were purified by RP-HPLC using the following conditions: 0.90 minutes in H_2O + 0.2% formic acid (FA), then linear gradient to 50% MeCN + 0.2% FA in 5.70 minutes at a flow rate of 0.25 mL/min.The purity of purified batches was again checked by UPLC-MS. Quantification was performed by dissolving the PNA obtained in 1 mL of water and measuring the absorbance at 260 nm by UV-Vis spectroscopy, using additive base contributions for the calculation of molar absorptivity (ε_{260}: T = 8600, C = 6600, A = 13700, G = 11700 $M^{-1}cm^{-1}$, Rho B = 23173 $M^{-1}cm^{-1}$).

PNA Characterization: UPLC-ESI-MS was carried out by using a Waters Acquity Ultra Performance LC with Waters Acquity SQ Detector and with ESI interface and equipped with a Waters Acquity UPLC BEH 300 (50x2.1 mm, 1.7 µm, C18) (UPLC1, 0.90 minutes in H_2O 0.2% FA, then linear gradient to 50% MeCN 0.2% FA in 5.70 minutes at a flow rate of 0.25 mL/min; UPLC2, 0.90 minutes in H_2O 0.2% FA, then linear gradient to 100% MeCN 0.2% FA in 5.70 minutes at a flow rate of 0.25 mL/min). PNA oligomers were purified with RP-HPLC using a Phenomenex Jupiter C18 (5 µm, 300 Å, 250x10 mm) (HPLC1, linear gradient from H_2O 0.1% TFA to 50% MeCN 0.1 % TFA in 30 minutes at a flow rate of 4.0 ml/min).

PNA1: Rho B-TTT CGT TAT TGC TCT TGA- Gly-NH_2, ε (260 nm): 191473 $M^{-1}cm^{-1}$, 14.6 % yield.
UPLC-MS (ESI): r.t. = 5.48 min, m/z: [M] Calcd 5340.3; Found 1335.9 $[M+4H]^{4+}$, 1069.0 $[M+5H]^{5+}$, 890.9 $[M+6H]^{6+}$, 763.9 $[M+7H]^{7+}$, 668.5 $[M+8H]^{8+}$, 594.4 $[M+9H]^{9+}$ (**Figure S1**).

PNA1-R8: R8- TTT CGT TAT TCT TGA-Gly-NH_2, ε (260 nm): 168300 $M^{-1}cm^{-1}$, 3.1 % yield.
UPLC-MS (ESI): r.t. = 2.64 min, m/z: [M] Calcd 6164.3; Found 1234.0 $[M+5H]^{5+}$, 1028.5 $[M+6H]^{6+}$, 881.7 $[M+7H]^{7+}$, 771.5 $[M+8H]^{8+}$, 686.0 $[M+9H]^{9+}$, 617.4 $[M+10H]^{10+}$, 561.4 $[M+11H]^{11+}$ (**Figure S2**).

PNA1-E8: E8- TTT CGT TAT TCT TGA-Gly-NH_2, ε (260 nm): 168300 $M^{-1}cm^{-1}$, 11 % yield.
UPLC-MS (ESI): r.t. = 3.0 min, m/z: [M] Calcd 5947.6; Found 1190.9 $[M+5H]^{5+}$, 992.6 $[M+6H]^{6+}$, 850.8 $[M+7H]^{7+}$, 744.7 $[M+8H]^{8+}$ (**Figure S3**).

PNA2: Rho B-AGT TAT CAC AGT ACT GTA-Gly-NH_2, ε (260 nm): 211873 $M^{-1}cm^{-1}$, 5.8 % yield.
UPLC-MS (ESI): r.t. = 5.04, 5.72, 6.57 min, m/z: [M] Calcd 5376.4; Found 1345.1 $[M+4H]^{4+}$, 1076.0 $[M+5H]^{5+}$, 897.1 $[M+6H]^{6+}$, 769.4 $[M+7H]^{7+}$, 673.6 $[M+8H]^{8+}$, 598.0 $[M+9H]^{9+}$ (**Figure S4**).

PNA2-R8: R8- AGT TAT CAC AGT ACT GTA-Gly-NH_2, ε (260 nm): 188700 $M^{-1}cm^{-1}$, 13.6 % yield.
UPLC-MS (ESI): r.t. = 2.69 min, m/z: [M] Calcd 6200.3; Found 1241.3 $[M+5H]^{5+}$, 1035.3 $[M+6H]^{6+}$, 887.4 $[M+7H]^{7+}$, 776.8 $[M+8H]^{8+}$, 690.8 $[M+9H]^{9+}$, 621.7 $[M+10H]^{10+}$ (**Figure S5**).

PNA2-E8: E8- AGT TAT CAC AGT ACT GTA-Gly-NH_2, ε (260 nm): 188700 $M^{-1}cm^{-1}$, 10.1 % yield.
UPLC-MS (ESI): r.t. = 2.91 min, m/z: [M] Calcd 5983.7; Found 1497.3 $[M+4H]^{4+}$, 1198.0 $[M+5H]^{5+}$, 998.4 $[M+6H]^{6+}$, 856.0 $[M+7H]^{7+}$, 749.1 $[M+8H]^{8+}$, 666.0 $[M+9H]^{9+}$ (**Figure S6**).

PNA3: Rho B-AGG GAT TCC TGG GAA AAC-Gly-NH_2, ε (260 nm): 221173 $M^{-1}cm^{-1}$, 1.2 % yield.
UPLC-MS (ESI): r.t. = 5.09 min, m/z: [M] Calcd 5451.4; Found 1363.8 $[M+4H]^{4+}$, 1091.1 $[M+5H]^{5+}$, 909.5 $[M+6H]^{6+}$, 779.8 $[M+7H]^{7+}$, 682.4 $[M+8H]^{8+}$ (**Figure S7**).

PNA3-R8: R8- AGG GAT TCC TGG GAA AAC-Gly-NH_2, ε (260 nm): 198000 $M^{-1}cm^{-1}$, 4.8 % yield.
UPLC-MS (ESI): r.t. = 2.50 min, m/z: [M] Calcd 6275.4; Found 1256.4 $[M+5H]^{5+}$, 1046.9 $[M+6H]^{6+}$, 897.7 $[M+7H]^{7+}$, 785.5 $[M+8H]^{8+}$, 698.4 $[M+9H]^{9+}$, 628.7 $[M+10H]^{10+}$ (**Figure S8**).

PNA3-E8: E8- AGG GAT TCC TGG GAA AAC-Gly-NH_2, ε (260 nm): 198000 $M^{-1}cm^{-1}$, 1 % yield.
UPLC-MS (ESI): r.t. = 2.86 min, m/z: [M] Calcd 6058.3; Found 1213.1 $[M+5H]^{5+}$, 1010.9 $[M+6H]^{6+}$, 866.7 $[M+7H]^{7+}$, 758.5 $[M+8H]^{8+}$(**Figure S9**).

Porous Silicon Nanoparticle Fabrication and Characterization (pSiNPs): PSi films were prepared by electrochemical perforation etching of a single crystal, (100)-oriented p-type silicon wafer in an electrolyte consisting of 3:1 (v:v) of 48% aqueous HF: absolute ethanol. The preparation followed a published "perforated etch" procedure[3]. Etching was carried out in a Teflon etch cell using a platinum coil counter electrode. Prior to preparation of the porous silicon layers, the wafer surface was cleaned using a sacrificial etch consisting of electrochemical anodization (60 sec, 46 mA/cm^2) in an electrolyte consisting of 3:1 (v:v) 48% aqueous HF : absolute ethanol, followed by ethanol rinse, then dissolution of the porous film with aqueous KOH (1 M). The wafer was rinsed with water, then ethanol. The etching waveform consisted of a square wave in which a lower value of current density of 46 mA/cm^2 was applied for 1.82 s, followed by an upper value of current density of 365 mA/cm^2 applied for 0.363 s (Keithley 2651A Sourcemeter power supply). The layered porous nanostructure was removed from the crystalline silicon substrate by application of current pulse of 3.7 mA/cm^2 for 250 s in an electrolyte consisting of 1:29 (v:v) of 48% aqueous HF: absolute ethanol. The freestanding pSi film was then fractured by ultrasonication (VWR International) in deionized water (7 mL) overnight and the resulting pSiNPs were dispersed in an aqueous solution of sodium tetraborate 0.8 mM. The resulting porous silicon nanoparticles were collected using centrifugation (15,000 rpm, 10 min, Eppendorf Centrifuge Model 5424R) and washed 3 times with ethanol.
The nanoparticles were characterized by attenuated total reflectance Fourier transform infrared (ATR-FTIR) spectra recorded using a Thermo Scientific Nicolet 6700 FTIR instrument fitted with a Smart iTR diamond ATR fixture. Dynamic light scattering (DLS) and zeta-potential of nanoparticles were determined using a Malvern Instruments Zetasizer Nano ZS90. Transmission electron microscope (TEM) images were obtained with a JEOL-1200 EX II 120 kV instrument. Nitrogen adsorption-desorption isotherms of the pSi microparticles were recorded at 77 K using a Micro-meritics ASAP 2020 instrument. Prior to the nitrogen adsorption experiment, the pSi microparticles were degassed under vacuum overnight. The surface area of the particles was determined using the BET (Brunauer-Emmett-Teller) method. The nanoparticles were stored in ethanol and periodically checked by DLS for integrity.

Preparation of calcium-silicate coated PNA-loaded pSiNPs (Ca-PNA-pSiNPS): A stock solution 4M in calcium chloride ($CaCl_2$) was prepared in RNAse-free water. The solution was centrifuged to remove any precipitates and stored at 4 °C before use. A solution of PNA 150 μM in water (50 μL) was mixed with 0.25 mg pSiNP dispersed in 200 μL of ethanol. To this mixture 250 μL of a $CaCl_2$ solution were added and the reaction was carried out for 45' under mixing. The nanoparticles were washed three times by centrifugation, first using deionized (DI) water (200 μL), then 70% ethanol, and finally absolute ethanol, and the first supernatant was recovered. The analysis of the supernatant was performed using a calibration curve registered in the same medium as the final supernatant solution (200 μL ethanol, 250 μL $CaCl_2$, 50 μL water). The loading was calculated by the difference between the starting PNA solution and the supernatant.

PNA Release: PNA-loaded Ca-pSiNPs (0.25 mg, n = 3) were dispersed in 1 mL of PBS (pH 7.4) and incubated at 37° with mild shaking. The supernatant containing released PNA was collected after centrifugation and the remaining pSiNPs were re-suspended in 1 mL of PBS at 1, 2, 4, 8, 24 and 48 h. PNA release was determined by measuring PNA concentration in the supernatants at each centrifugation step by means of UV-Vis spectroscopy at λ = 260 nm.

Cell Culture: The human bronchial epithelial IB3-1 cell line was cultured in a humidified atmosphere of 5% CO_2/air in LHC-8 medium (Gibco, Thermo Fischer Scientific, Waltham, Massachusetts, USA) supplemented with 5% fetal bovine serum (FBS, Biowest, Nuaillé, France) in the absence of gentamycin. To verify the effect on proliferation, cell growth was monitored by determining the cell number/ml using a Z2 Coulter Counter (Coulter Electronics, Hialeah, FL, USA).

Cell Transfection with Nanoparticles: Suspensions of nanoparticles were equilibrated at room temperature for 10 min, centrifuged at 10,000 rpm to completely remove ethanol and resuspended in DPBS. Twelve hours before the procedure, IB3-1 cells were seeded at 50% of confluence and then transfected with nanoparticles functionalized with rhodamine-labeled PNAs, at a PNA final concentration of 2 or 4 µM. Cells were incubated in humidified atmosphere of 5% CO_2/air for 24 hours until FACS analysis.

FACS Analysis: Uptake of rhodamine-labeled PNAs was evaluated, after 24 hours from transfection, using FACS Canto II (BD, Becton Dickinson, Franklin Lakes, New Jersey, USA), in the PE (Phycoerythrin) channel. Cells were detached with trypsin and collected by centrifugation at 1,200 rpm for 10 minutes at room temperature, washed with DPBS 1X, re-suspended in 200 µL of DPBS 1X and analyzed by FACS analysis. For each sample, 10,000 events were acquired, and data analysis was performed using BD FACSDiva Software (BD, Becton Dickinson, Franklin Lakes, New Jersey, USA).

Cell imaging acquisition: Internalization of fluorescently labelled PNAs loaded into nanoparticles was evaluated using BioStation IM (Nikon, Minato, Tokyo, Japan). Cells were pre-treated with Hoechst 33342 dye, at final concentration of 5 µg/µL to identify nucleus position into cells. Cells were incubated with Hoechst diluted in culture medium for 20 min at 37°C, in the dark, washed twice with DPBS and then new medium was added. Cells were treated with a) porous silicon nanoparticles, b) rhodamine B (Rho) conjugated PNAs or c) nanoparticles loaded with rhodamine conjugated PNAs, for 48 h as previously described. Images were taken after 48 hours of incubation using DAPI filter (461 nm) to visualize nuclei and 570 nm filter (TRITC) to visualize rhodamine B conjugate molecules. Two different magnifications were employed x40 and x80. Pictures are presented as merge of live, DAPI and TRITC images or as merge of live and TRITC images.

RNA Extraction: Transfected cells were detached by trypsinization and centrifuged at 1,200 rpm for 10 min at 4 °C, washed with cold DPBS 1X and lysed with 1 mL of Tri-Reagent (Sigma Aldrich, St. Louis, Missouri, USA), according to manufacturer’s instructions. Isolated RNA was washed once with cold 75% ethanol, air-dried and dissolved in nuclease-free water before use.

RT-qPCR: To verify miRNAs content within cells, obtained RNA was reverse transcribed using TaqMan MicroRNA Reverse Transcription Kit (Applied Biosystems, Foster City, CA, USA) and miRNA specific stem-loop primers (hsa-miR-101-3p, ID: 002253; hsa-miR-145-5p, ID: 002278; hsa-miR-335-5p, ID: 000546). Reverse transcription quantitative polymerase-chain reaction (RT-qPCR) was performed according to the manufacturer’s protocol. All RT reactions, including RT-minus controls and no-template controls, were run in duplicate using the CFX96 Touch Real-Time PCR Detection System (BioRad, Hercules, CA, USA) using TaqMan Universal PCR Master Mix, no AmpErase UNG 2x (Applied Biosystems, Foster City, CA, USA). The relative expression was calculated using the comparative cycle threshold method (ΔΔCT) and using simultaneously U6 snRNA (hsa U6 snRNA, ID:001973) and hsa-let-7c (hsa-let-7c, ID:000379) as endogenous controls.

2. Supporting figures

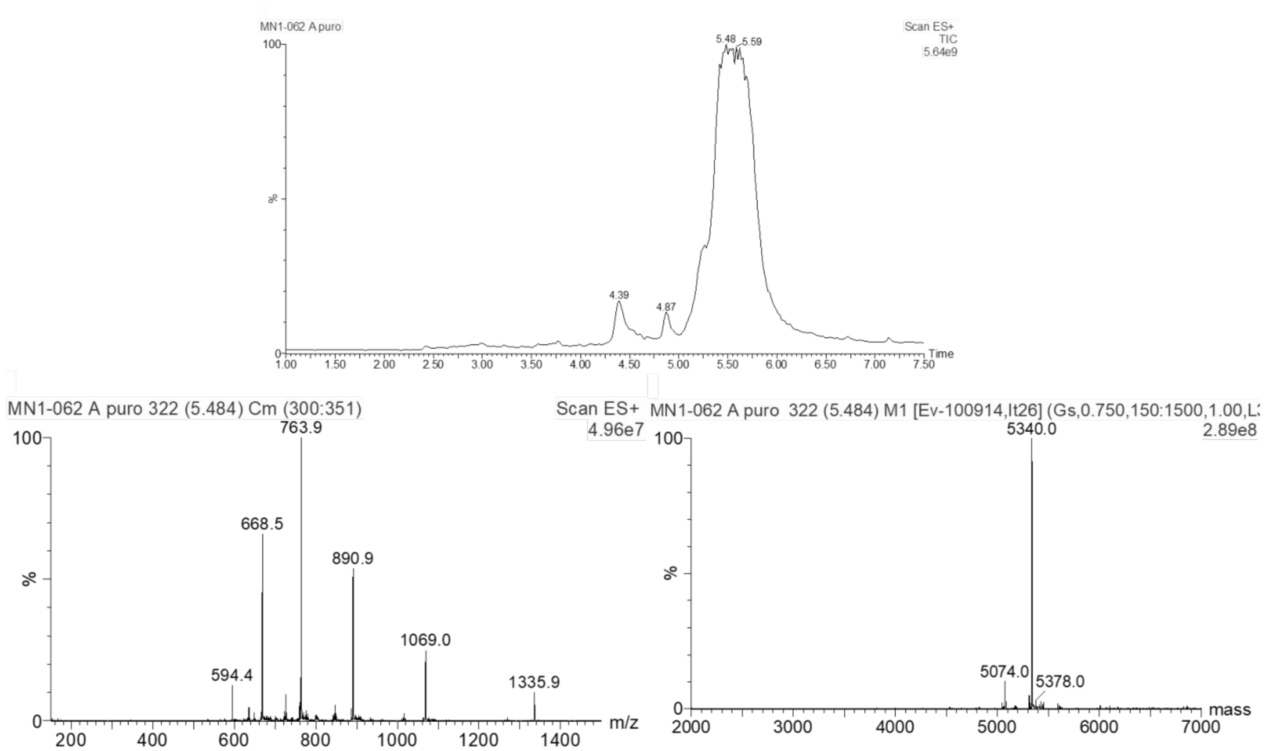

Figure S1. UPLC-MS chromatogram of pure PNA1 (top) and ESI-MS spectrum for the peak at 5.48 min with the corresponding mathematic deconvolution of the multicharged signals (bottom).

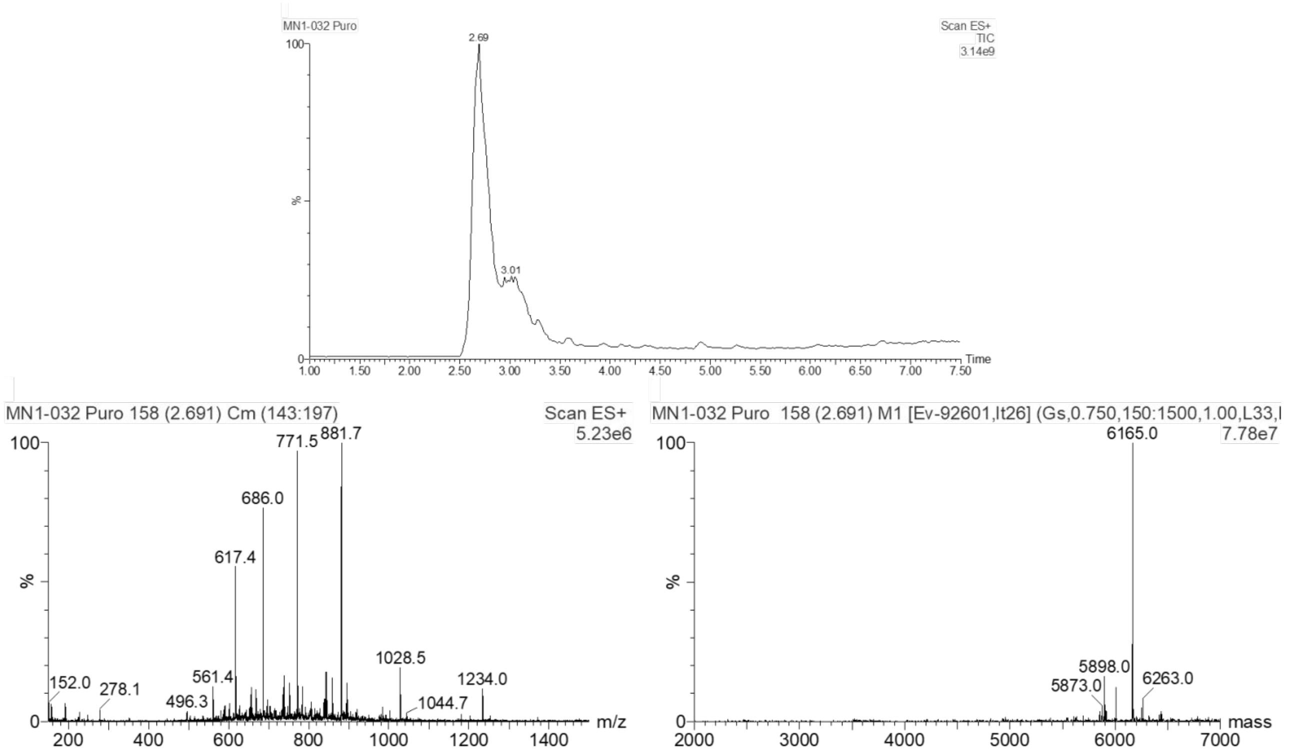

Figure S2. UPLC-MS chromatogram of pure PNA1-R8 (top) and ESI-MS spectrum for the peak at 2.69 min with the corresponding mathematic deconvolution of the multicharged signals (bottom).

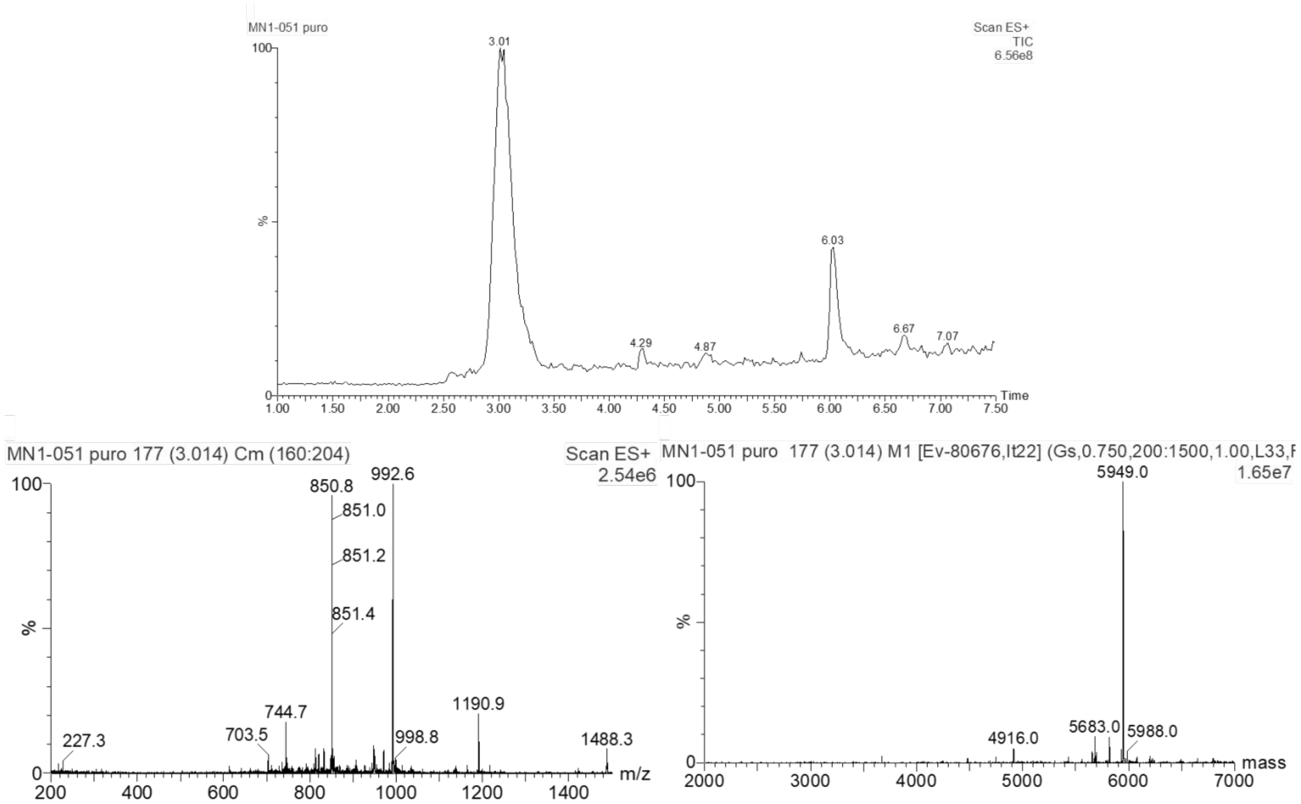

Figure S3. UPLC-MS chromatogram of pure PNA1-E8 (top) and ESI-MS spectrum for the peak at 3.01 min with the corresponding mathematic deconvolution of the multicharged signals (bottom).

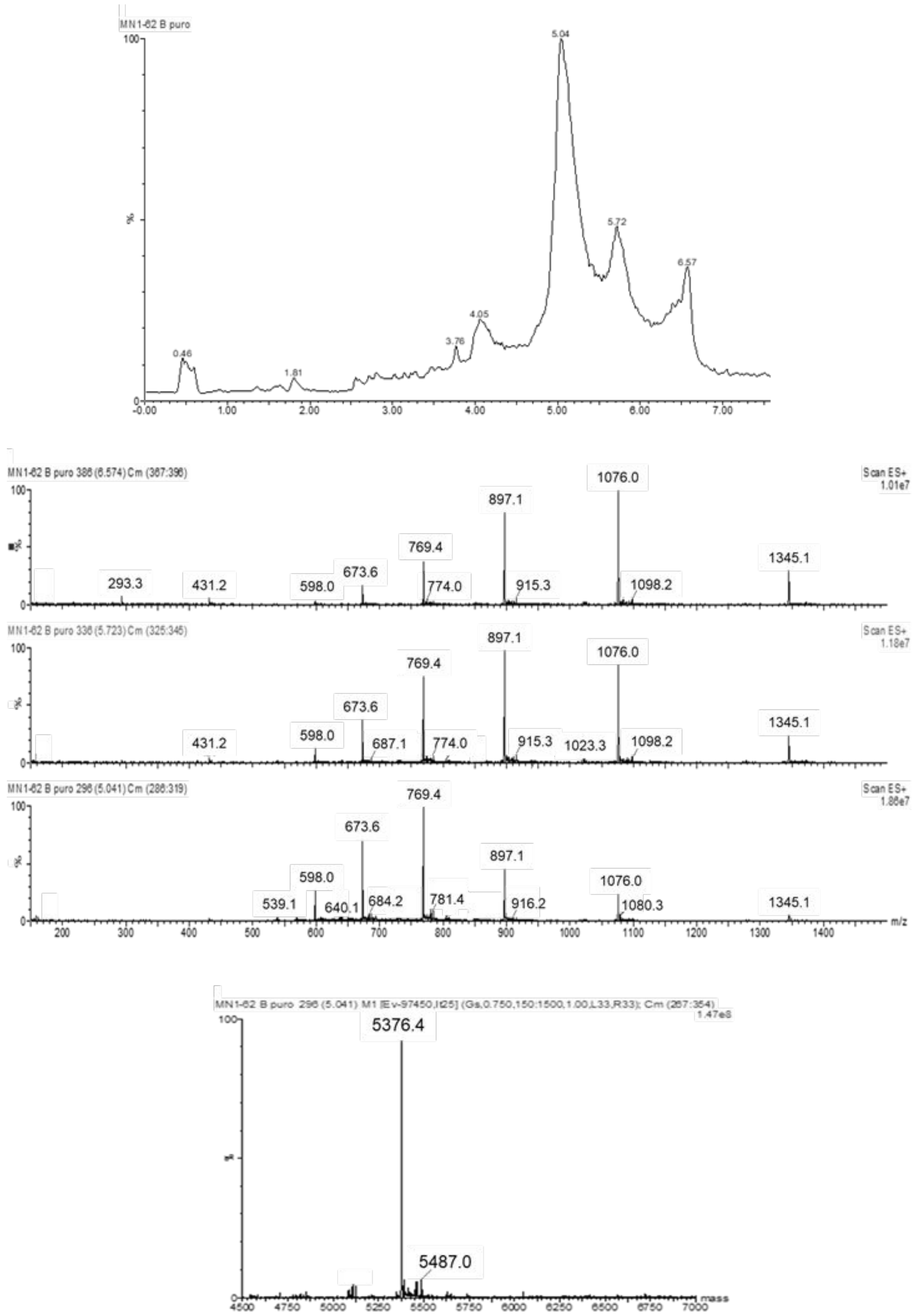

Figure S4. UPLC-MS chromatogram of pure PNA2 (top) and ESI-MS spectrum (central) for the peak at 5.04 min with the corresponding mathematic deconvolution of the multicharged signals (bottom).

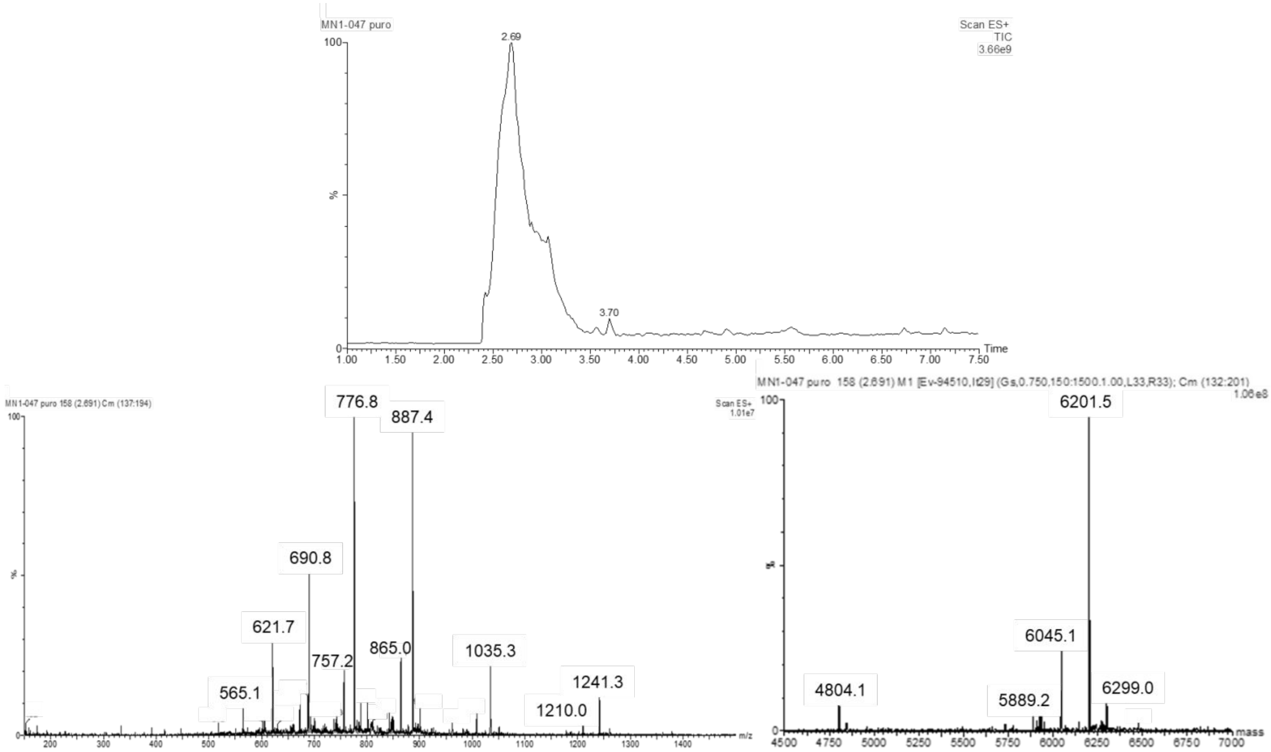

Figure S5. UPLC-MS chromatogram of pure PNA2-R8 (top) and ESI-MS spectrum for the peak at 2.69 min with the corresponding mathematic deconvolution of the multicharged signals (bottom).

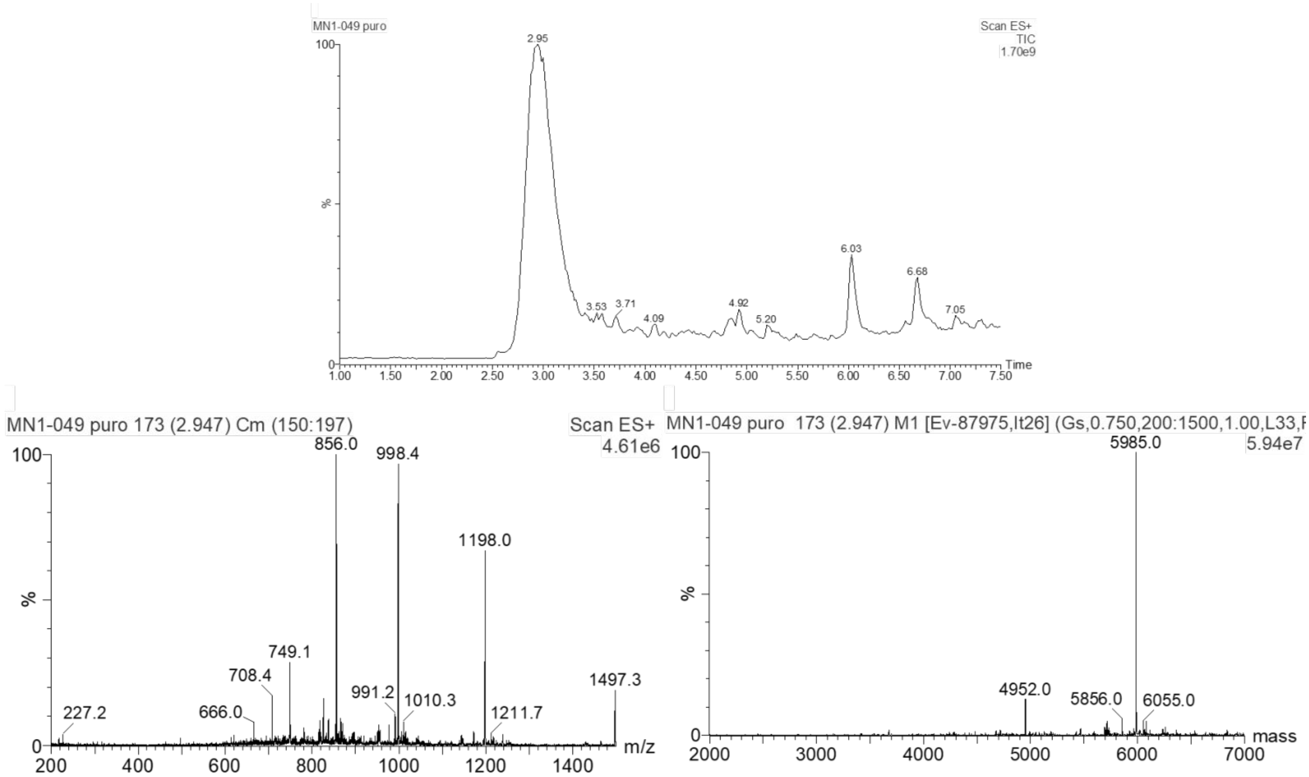

Figure S6. UPLC-MS chromatogram of pure PNA2-E8 (top) and ESI-MS spectrum for the peak at 2.95 min with the corresponding mathematic deconvolution of the multicharged signals (bottom).

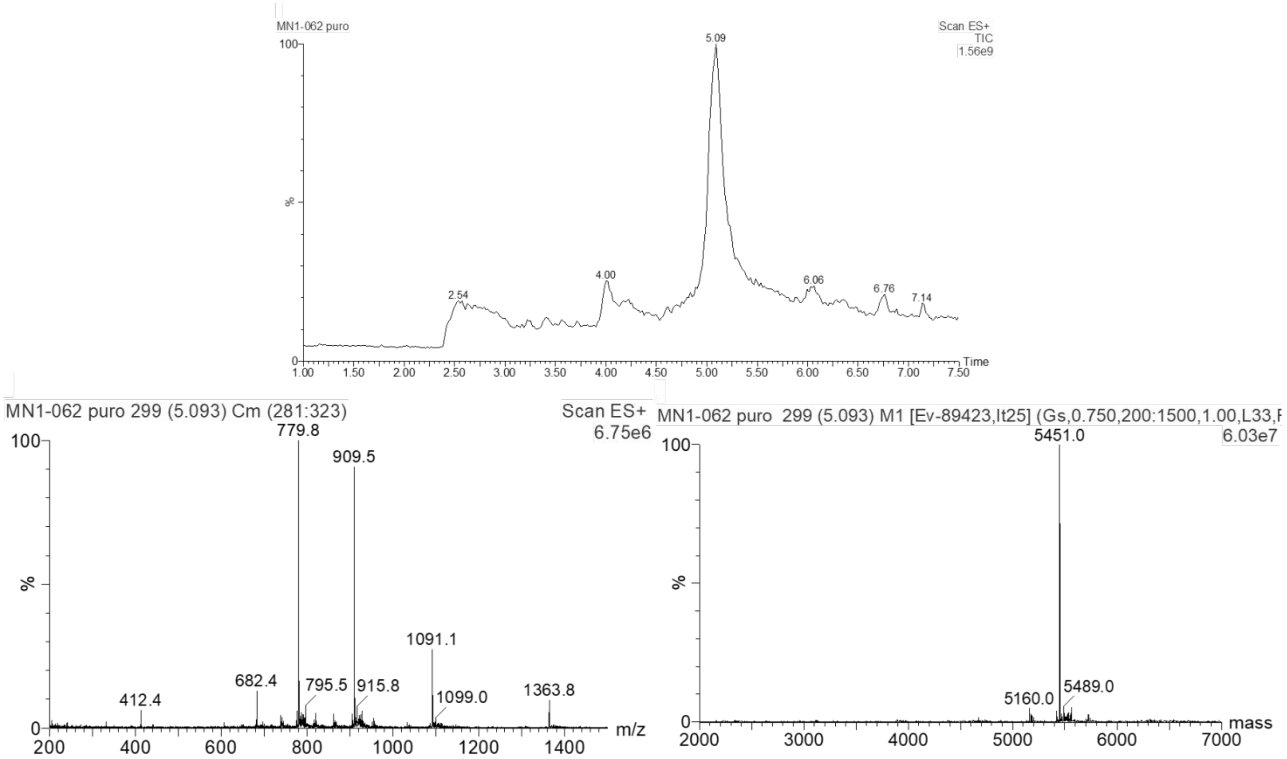

Figure S7. UPLC-MS chromatogram of pure PNA3 (top) and ESI-MS spectrum for the peak at 5.09 min with the corresponding mathematic deconvolution of the multicharged signals (bottom).

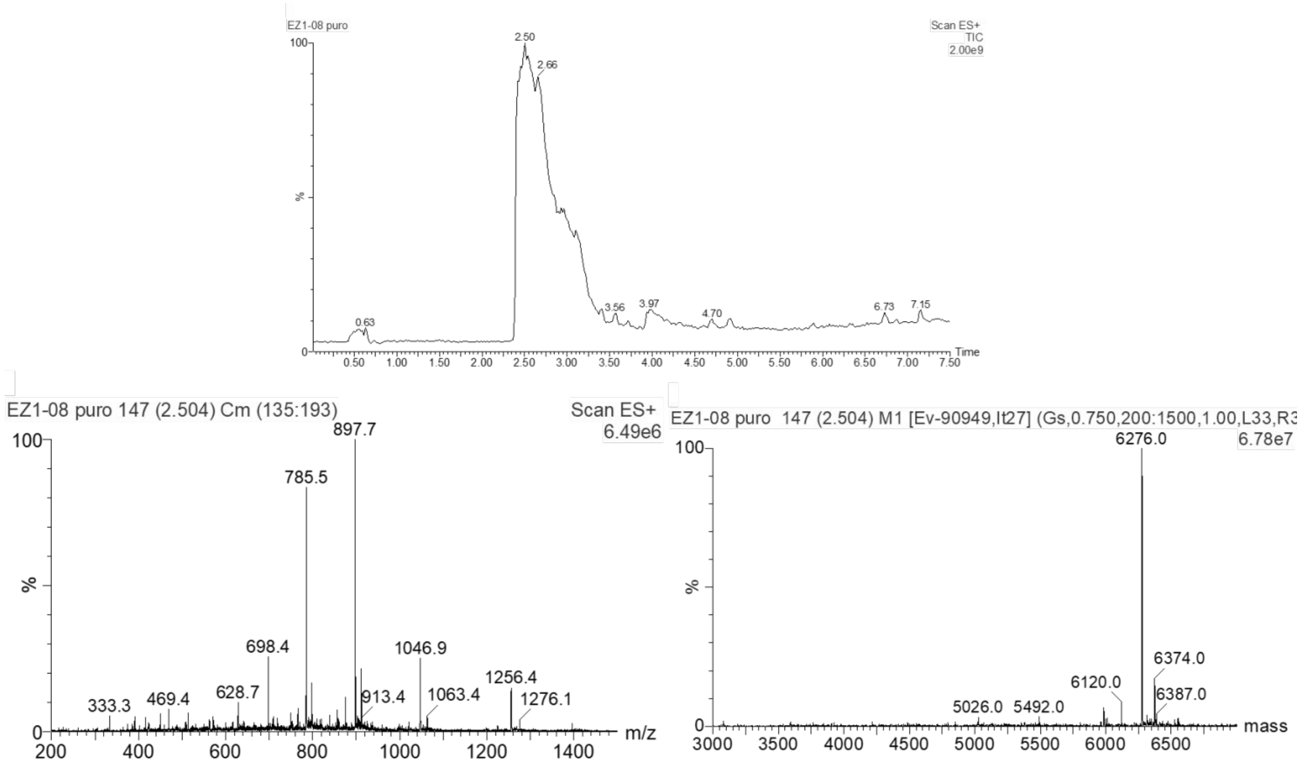

Figure S8. UPLC-MS chromatogram of pure PNA3-R8 (top) and ESI-MS spectrum for the peak at 2.50 min with the corresponding mathematic deconvolution of the multicharged signals (bottom).

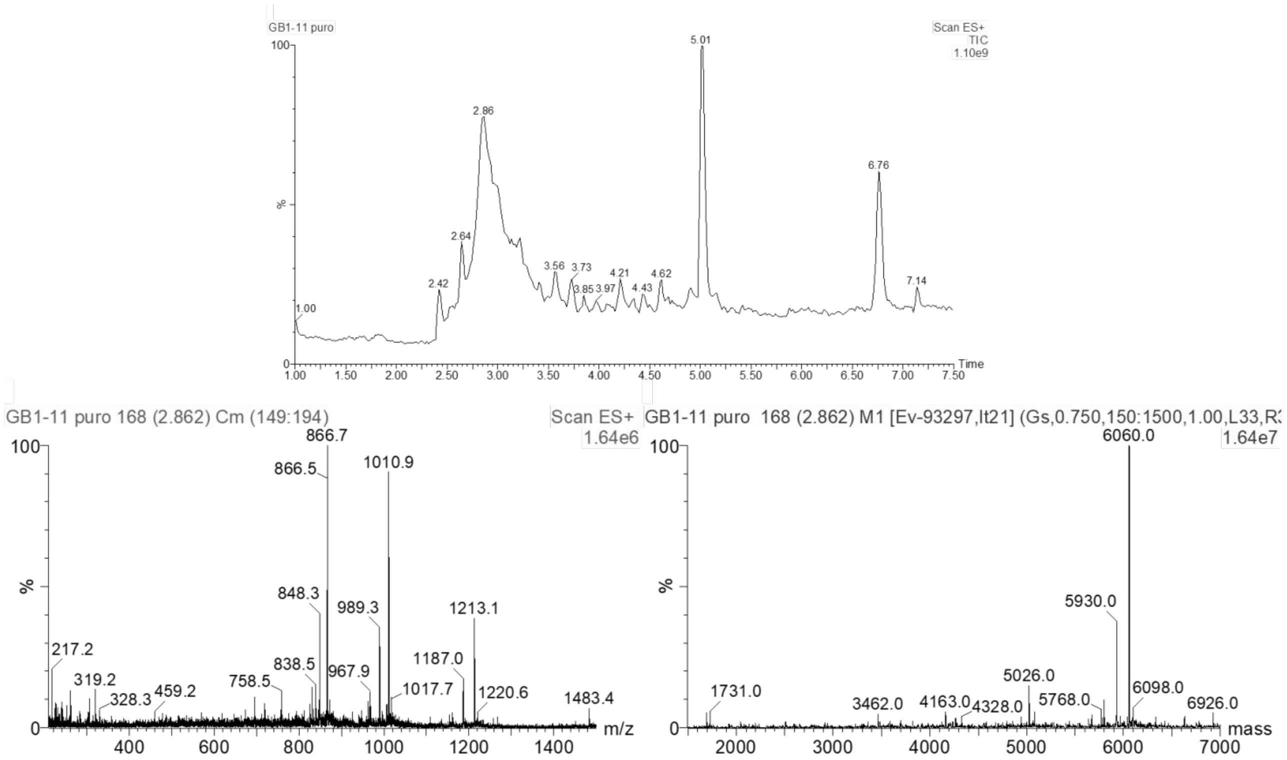

Figure S9. UPLC-MS chromatogram of pure PNA3-E8 (top) and ESI-MS spectrum for the peak at 2.86 min with the corresponding mathematic deconvolution of the multicharged signals (bottom).

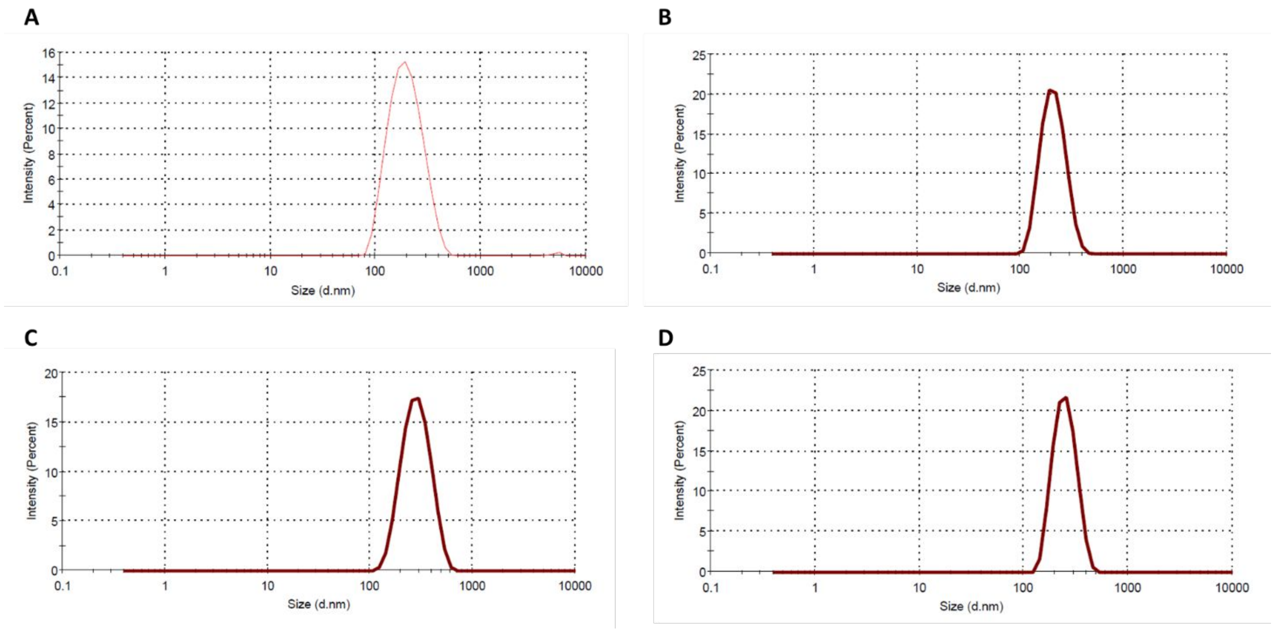

Figure S10. Intensity weighted size distribution from dynamic light scattering of freshly etched pSiNPs (PDI: 0.160) (A), and after loading with negatively charged PNA 3 (PDI: 0.228) (B), positively charged PNA 3 (PDI: 0.180) (C) and neutral PNA 2 (PDI: 0.226) (D).

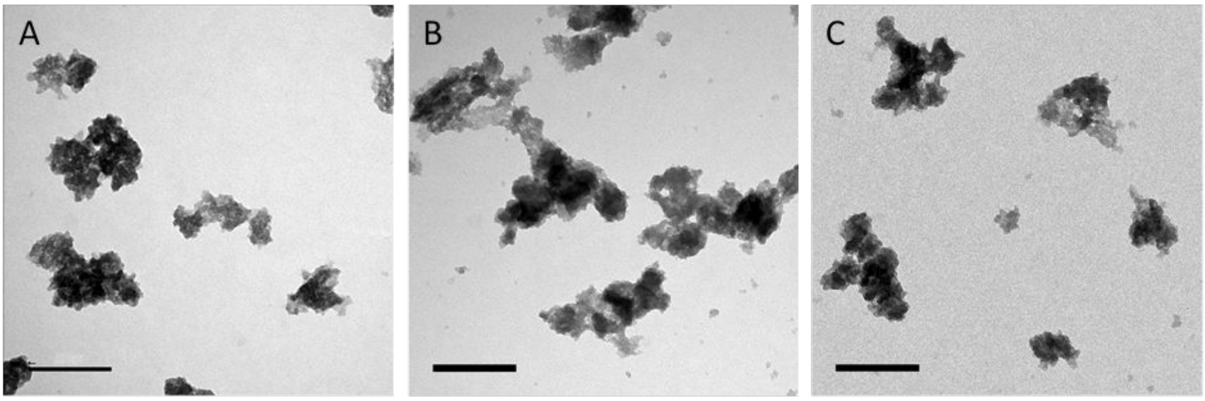

Figure S11. Transmission electron microscope images of Ca-pSiNPs-PNA3-E8 (A), Ca-pSiNPs-PNA3-R8 (B) and Ca-pSiNPs-PNA2 (C). Scale bar = 200 nm for (A), (B) and (C).

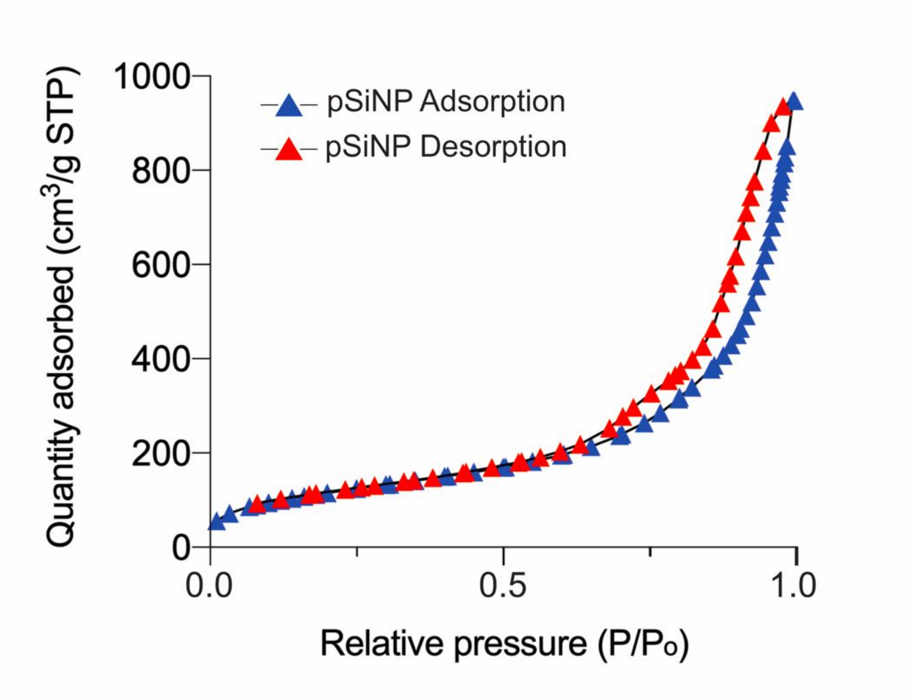

Figure S12. Cryogenic nitrogen adsorption−desorption isotherm of bare pSiNPs. The average pore size was found to be 14.5 nm with a surface area of 370.5 m²/g.

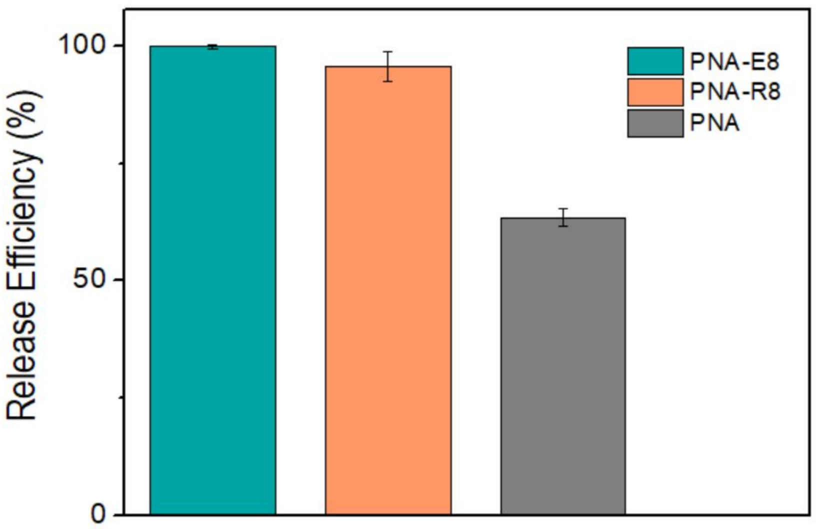

Figure S13. Total release efficiency of three distinct PNAs from pSiNPs in PBS at 37°C. At the end point of the release studies, 98.7 % ± 0.4 of total loaded PNA-E8 was released from the pSiNPs, 96 % ± 3 of total loaded PNA-R8 was released from the pSiNPs, 64 % ± 2 of total loaded PNA was released from the pSiNPs.

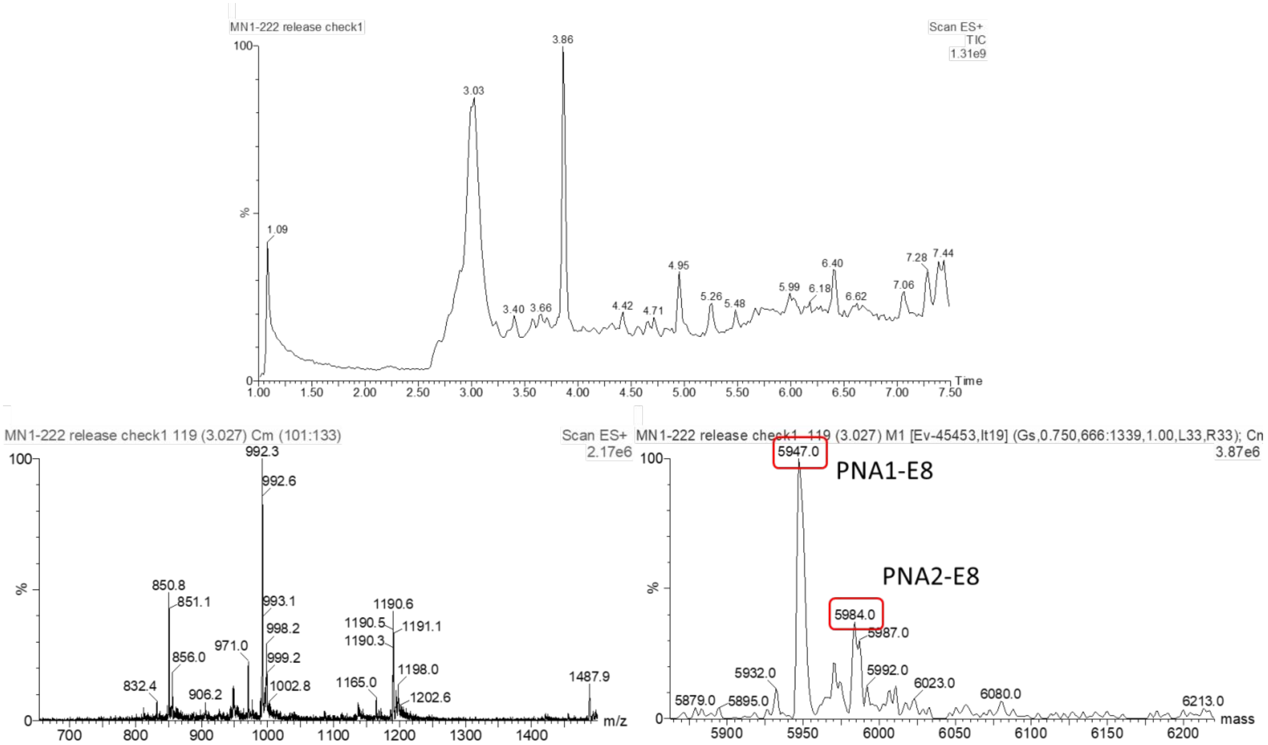

Figure S14. UPLC-MS chromatogram of supernatant (top) and ESI-MS spectrum for the peak at 3.03 min with the corresponding mathematical deconvolution of the multicharged signals that shows the mass of the two loaded PNAs (bottom).

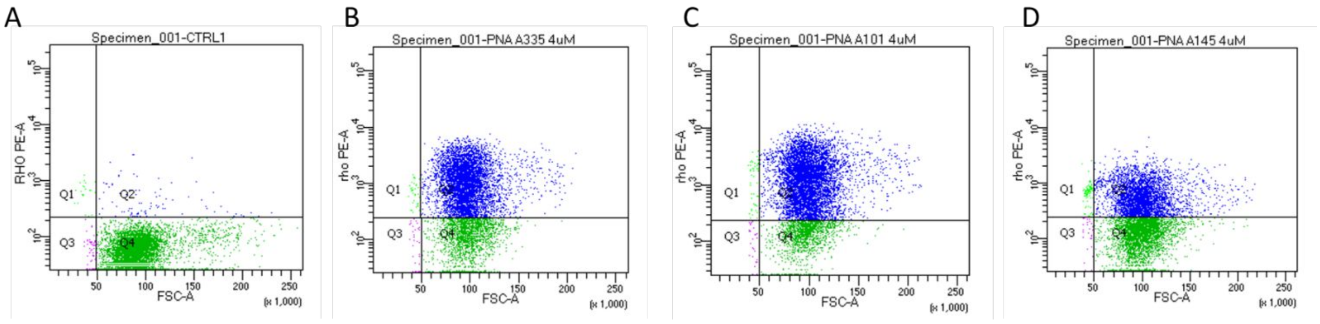

Figure S15. Flow cytometry profiles of IB3-1 cells in absence of treatment (A) and treated with doses of PNAS-loaded pSiNPs corresponding to a theoretical concentration of PNA in the incubation well of 4 μM of PNA 1 (B), PNA 2 (C) and PNA 3 (D).

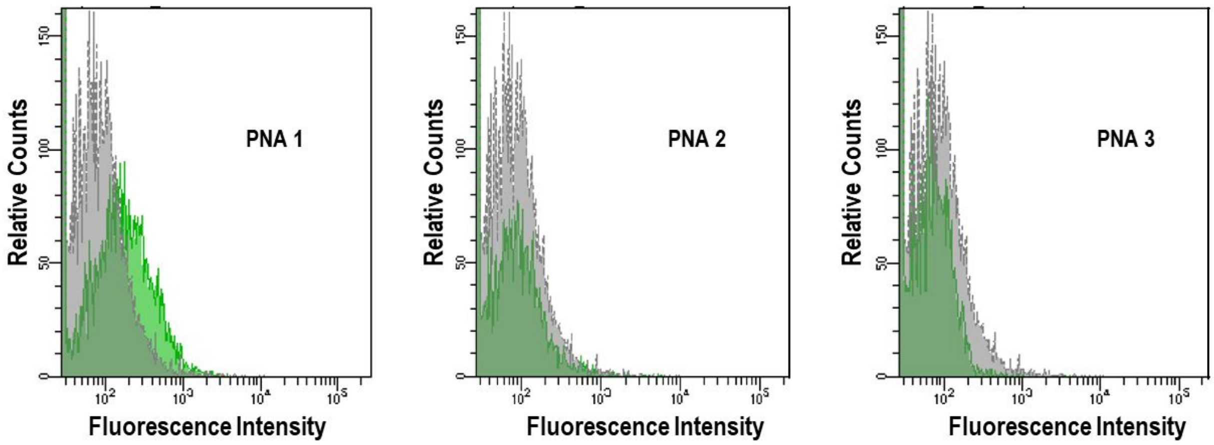

Figure S16. Flow cytometry graphs of untreated IB3-1 cells (grey) and IB3-1 cells treated with only PNAs at concentration 4μM (green).

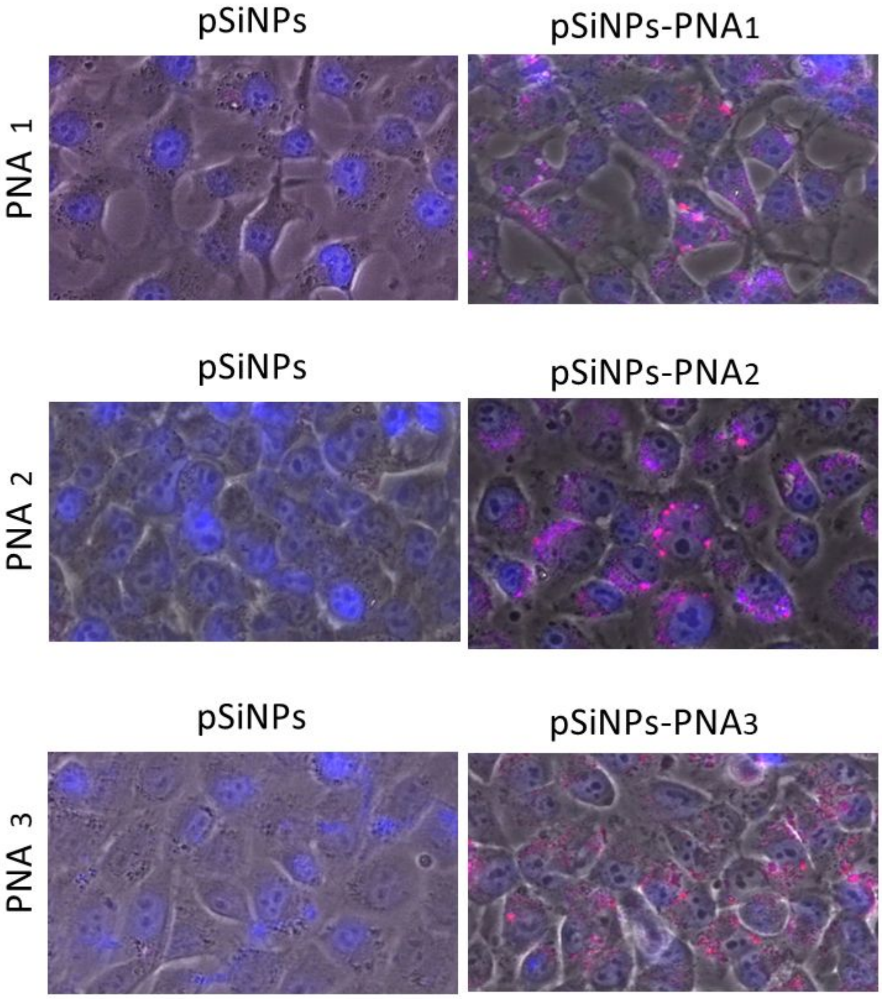

Figure S17. Internalization of pSiNP-PNA1, pSiNP-PNA2 and pSiNP-PNA3 complexes evaluated using BioStation IM (Nikon, Minato, Tokyo, Japan). Cells were either treated with pSiNPs or pSiNP-PNA1 complexes, as indicated. Pictures are presented as merge of live, DAPI and TRITC images.

Supporting References

(1) Manicardi, A.; Gambari, R.; de Cola, L.; Corradini, R. Preparation of Anti-MiR PNAs for Drug Development and Nanomedicine. In *DNA Nanotechnology: Methods and Protocols*; Zuccheri, G., Ed.; Springer New York: New York, NY, 2018; pp 49–63.

(2) Mejia-Ariza, R.; Rosselli, J.; Breukers, C.; Manicardi, A.; Terstappen, L. W. M. M.; Corradini, R.; Huskens, J. DNA Detection by Flow Cytometry Using PNA-Modified Metal–Organic Framework Particles. *Chem. – A Eur. J.* **2017**, *23* (17), 4180–4186.

(3) Qin, Z.; Joo, J.; Gu, L.; Sailor, M. J. Size Control of Porous Silicon Nanoparticles by Electrochemical Perforation Etching. *Part. Part. Syst. Charact.* **2014**, *31*, 252–256.
